# Comparative promoter region analysis powered by CORG

**DOI:** 10.1186/1471-2164-6-24

**Published:** 2005-02-21

**Authors:** Christoph Dieterich, Steffen Grossmann, Andrea Tanzer, Stefan Röpcke, Peter F Arndt, Peter F Stadler, Martin Vingron

**Affiliations:** 1 Computational Molecular Biology Department, Max Planck Institute for Molecular Genetics, Ihnestrasse 73, 14195 Berlin, Germany; 2 Institute for Theoretical Chemistry and Structural Biology, University of Vienna, Währingerstrasse 17, A-1090 Wien, Austria; 3 Bioinformatics Group, Department of Computer Science, University of Leipzig, Kreuzstraße 7b, D-04103 Leipzig, Germany

## Abstract

**Background:**

Promoters are key players in gene regulation. They receive signals from various sources (e.g. cell surface receptors) and control the level of transcription initiation, which largely determines gene expression. In vertebrates, transcription start sites and surrounding regulatory elements are often poorly defined. To support promoter analysis, we present CORG , a framework for studying upstream regions including untranslated exons (5' UTR).

**Description:**

The automated annotation of promoter regions integrates information of two kinds. First, statistically significant cross-species conservation within upstream regions of orthologous genes is detected. Pairwise as well as multiple sequence comparisons are computed. Second, binding site descriptions (position-weight matrices) are employed to predict conserved regulatory elements with a novel approach. Assembled EST sequences and verified transcription start sites are incorporated to distinguish exonic from other sequences.

As of now, we have included 5 species in our analysis pipeline (man, mouse, rat, fugu and zebrafish). We characterized promoter regions of 16,127 groups of orthologous genes. All data are presented in an intuitive way via our web site. Users are free to export data for single genes or access larger data sets via our DAS server . The benefits of our framework are exemplarily shown in the context of phylogenetic profiling of transcription factor binding sites and detection of microRNAs close to transcription start sites of our gene set.

**Conclusion:**

The CORG platform is a versatile tool to support analyses of gene regulation in vertebrate promoter regions. Applications for CORG cover a broad range from studying evolution of DNA binding sites and promoter constitution to the discovery of new regulatory sequence elements (e.g. microRNAs and binding sites).

## Background

Comparative sequence analysis has been a powerful tool in bioinformatics for addressing a variety of issues. Applications range from grouping of sequences (e.g. protein sequences into families) to *de novo *pattern discovery of functional signatures.

Speaking of gene regulation, it has been known for a long time that there is considerable sequence conservation between species in non-coding regions of the genome. A comprehensive explanation of this observation is still elusive. However, sequence conservation within promoter regions of genes often stems from transcription factor binding sites that are under selective pressure (see [1] for a review and [2] for a systematic assessment of binding site conservation in man and mouse comparisons).

Conserved sequence elements of other types have recently caught much attention. Not all non-coding conserved DNA in the vicinity of a gene's transcription start site necessarily functions at the level of transcriptional regulation. For example, most known methylation-guide snoRNAs are intronencoded and processed from transcripts of housekeeping genes [3]. A few microRNAs are apparently linked to protein coding genes, most notably *mir-10 *and *mir-196 *which are located in the (short) intergenic regions in the *Hox *gene clusters of vertebrates [4-7].

A second class of conserved sequence elements exert their function as regulatory motifs in the untranslated region (UTR) of the primary transcript or the mature mRNA. The UTRsite database [8], for example, lists about 30 distinct functional motifs including the Histone 3'UTR stem-loop structure (HSL3) [9], the Iron Responsive Element (IRE) [10], the Selenocysteine Insertion Sequences (SECIS) [11], and the Internal Ribosome Entry Sites (IRES) [12]. Most of these elements are contained in CORG since short intergenic regions or introns upstream of the translation start site are entirely covered by our definition of an upstream region.

### Phylogenetic footprinting

The CORG framework aims at detecting and describing regulatory elements that are proximal to the transcription start site. In this context, the comparison of upstream regions of orthologous genes is particularly valuable. This concept is called "phylogenetic footprinting" and an overview of this approach can be found in [13].

Phylogenetic footprinting in a strict sense is carried out on orthologous promoter regions. Local sequence similarities can then be directly interpreted as related regions harboring conserved functional elements. We denote these similarities as Conserved Non-coding Blocks (CNBs).

#### Multi-species sequence conservation

Comparative approaches gain power from the inclusion of sequences from more than two species [14]. Multi-species comparisons help to increase specificity at the expense of intra-species sensitivity since supporting evidence (conservation) stems from many observations. To give an example, Man-mouse-rat comparisons enhance the detection of transcription factor binding sites since the rat genome is more divergent from the mouse genome than anticipated [15]. A nice property of vertebrate microRNAs is the high degree of sequence conservation which is found in alignments of man, mouse and fish microRNAs [16]. Both types of comparisons are available in CORG. In CORG, we consider cross-species conservation between promoter regions from 5 vertebrate genomes, namely *Homo sapiens*, *Mus musculus*, *Rattus norvegicus*, *Danio rerio and Fugu rubripes*. Multiple alignments are built from pairwise CNBs as described in the subsequent section.

## Construction and content

### Groups of orthologous genes

In this work, we take a gene-centered view of phylogeny. Homology among proteins and thus genes is often concluded on the basis of sequence similarity. The EnsEMBL database [17] allows to distinguish orthologous from merely homologous genes by taking information on conserved synteny into account. We employed single linkage clustering on the graph of EnsEMBL orthologous gene pairs to define the CORG gene groups.

### Genomic mapping of validated promoter regions

Various recent experimental efforts supply information about the position of transcriptional start sites in the human and mouse genome. Table 1 gives an overview on the resources that were employed in CORG.

**Table 1:** Resources for validated transcription start sites

**Database name**	**Features**
Eukaryotic promoter database (EPD) [44]	The Eukaryotic promoter database is the smallest in size, but largely consists of manually curated entries.
DataBase of Transcriptional Start Sites (DBTSS) [45]	The DBTSS contains reliable information on the transcriptional start sites for man and mouse promoters. They exploit the oligo-capping technique to enrich their pool of clones for full-length 5'-to-3' cDNAs
H-Invitational Database (H-InvDB) [46]	H-InvDB is an international effort to integrate annotation of 41,118 full-length human cDNA clones that are currently available from six high throughput cDNA sequencing projects.
FANTOM 2 (RIKEN) [47]	The RIKEN consortium presented the FANTOM collection of RIKEN full-length cDNA clones. FANTOM stands for Functional Annotation of Mouse cDNA clones.
The Reference Sequence project (RefSeq) [48]	The Reference Sequence project aims to provide a comprehensive, integrated, non-redundant set of sequences, including full-length transcripts (mRNA)

Some repositories offer genomic coordinates for their start site entries. Existing genomic mapping information was incorporated unless the underlying genome assembly build differed. The remaining data were projected onto the genome with SSAHA (Sequence Search and Alignment by Hashing Algorithm), a rapid near-exact alignment algorithm [18].

### Sequence retrieval

The notion of "promoter region" deserves some further explanation in the context of our approach. Typically, though not exclusively, we expect conserved regulatory regions to appear in the vicinity of the transcription start site of a gene. Since we do not know the precise location of the start of transcription for each and every gene, we chose to compare the sequence regions upstream of the start of translation from orthologous genes. If verified transcription start sites are known, we define a sequence window that is large enough to hold both, translation and transcription start sites, plus 5 kB upstream sequence. In case we lack this information, our observations on known transcription start sites indicate that most promoter regions should be captured in a sequence window of 10 kb size (Additional File 1). The size of a promoter region may be bounded by the size of the corresponding intergenic region. If an annotated gene happens to lie within the primary sequence window, the promoter region is shortened to exclude exonic sequence.

**Figure 1:**
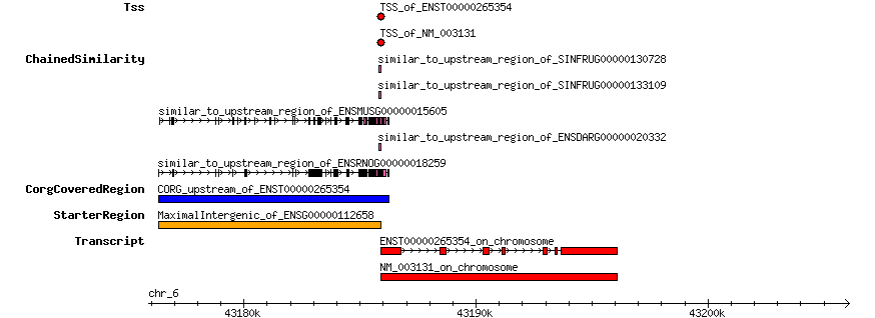
**Genomic context of human SRF. **This image is displayed after the user selected a gene identifier on the search page. It provides the user with the genomic context of the selected gene. Known and predicted transcription start sites are shown as labelled red dots. Local similarities to homologous regions from other species are shown as connected purple boxes. Blue bars depict all upstream regions as contained in CORG. The structure of the corresponding EnsEMBL transcripts as well as the extent of RefSeq transcripts is show in the bottom track.

### Detection of pairwise local sequence similarities

Significant local sequence similarities (phylogenetic footprints) in two sequences are computed with an implementation of the Waterman-Eggert algorithm. We have already given an account of the algorithm and statistics in [19,20]. The underlying alignment scoring scheme is the general reversible model [21]:



where *Q *is the transition rate matrix. We left out the elements on the diagonal, which are constrained by the requirement that the sum of all elements in a row equals zero.

The *π*_*i *_are the stationary nucleotide frequencies, their sum is constrained to be one. Although the two genomes under consideration are in general not in their stationary state with respect to the substitutional process we take the mean  of the two observed nucleotide frequencies, , to be the best estimate of the stationary base composition.

From other studies we have further knowledge about the relative rates between transversions, the transition A:T→G:C, and the transition G:C→A:T, which occur in roughly in the ratio 1:3:5 along vertebrate lineages [22]. These ratios of rates would generate sequences with 40% GC in their stationary state. To accommodate the observed nucleotide frequencies *π*_*i *_we have to allow for deviation from those ratios. We do this by choosing for example *α *∝ (*R*(*A *→ *T*)/*π*_*T *_+ *R*(*T *→ *A*)/*π*_*A*_)/2, where *R*(*i *→ *j*) is either 1, 3, or 5 depending on the process under consideration. At the end we scale the matrix *Q*, such that the PAM distance [23] of the substitution model equals the observed degree of divergence between the two species under comparison.

Since we were mainly interested in highly conserved regulatory elements, we demanded an average similarity level at least as high as the average exon conservation between the species under comparison.

The score for aligning two nucleotides *i *and *j *is then *s*(*i*, *j*) = log(*P*(*i*, *j*)/(*π*_*i*_*π*_*j*_)) where *P*(*i*, *j*) is the probability of finding the pairing of *i *and *j *under the above substitution model [21].

### Joining pairwise into multiple alignments

All CNBs from pairwise sequence alignments are split up into groups as defined by gene homology. For each group a graph *O *= (*V*, *E*) with vertices *V *and edges *E *is constructed, which represents the species-internal overlap of CNBs on the genomic coordinate level. Each vertex *a *∈ *V *represents a footprint, which is a pairwise local alignment between two species. An undirected edge is placed between two vertices if the corresponding CNBs have only one species in common and show an overlap of at least 10 bp on the sequence level.

In our graph *O*, cliques of minimal size three are detected with an implementation of the Bron-Kerbosh algorithm [24]. Only those cliques are selected whose species count is equal to their size. This move prohibits the emergence of multiple alignments by similarity of multiple short CNBs to a single long CNB. Multiple alignments are then computed based on all cliques that meet the outlined criteria. We chose to employ the multiple alignment method of [25] who applies partial order graphs (POG) to the multiple alignment problem.

Partial order graphs belong to the class of directed acyclic graphs (DAGs). A DAG is a graph consisting of a set of nodes *N *and edges *E*, which are one-way edges and form no cycles.

The multiple alignment problem is then reduced to to subsequent alignment steps of individual sequences to a growing multiple alignment graph. If the sequences to be aligned share substantial sequence similarity, the number of bifurcation points within the POG stays low and allows rapid computation of the multiple alignment.

Alignment results are subsequently trimmed to encompass the leftmost and rightmost ungapped block of at least 6 nucleotides.

### Annotation of promoter regions

#### Exon detection with assembled EST clusters

Promoter regions in CORG always extend upstream from the most downstream coding start (ATG). As a consequence, promoter regions may contain exons that are not translated. Our way of detecting such exons is a similarity search of man-mouse footprints versus *GENENEST *[26], a database of assembled EST clusters. Database searches are carried out for human and mouse footprints with the BLASTN program [27]. An E-value cut-off of 10^-4 ^is applied.

#### Annotation with predicted binding sites

The TRANSFAC database [28] is a repository of experimentally verified binding site sequences and representations thereof. These representations are used for querying the collection of man-mouse CNBs for known binding site patterns.

Potential binding sites are detected with TRANSFAC weight matrices by the method of [29]. Here, the intuition is that there are two random models for a given sequence *S*: one is given by the signal profile *F *and the other one by the background model *B*. Under both models the distribution of weight matrix scores can conveniently be calculated by convolution, since the score is a sum of independent random variables. Probability mass distributions of P_*F *_(Score(*S*)) as well as P_*B*_(Score(*S*)) can be computed by dynamic programming if column scores are reasonably discretized. This allows a fine tuning of the proportions of false positives and negatives for each TRANSFAC weight matrix. Both error levels were set to be equal. All details are given in [29].

## Utility and discussion

We now present an overview of the web interface of the database and several example applications.

### Interface

The CORG database is accessible via its home page  and offers a redesigned web interface. From the search page one can quickly jump to gene loci via EnsEMBL or other standard identifers (e.g. HUGO symbol, LocusLink identifier, ...). The search query is processed according to the chosen reference source and a list of all matching database entries is returned to the user. This list serves as a springboard to a summary page where the genomic context of the selected gene and its similarities to other upstream regions is visualized as in Figure 1.

Pairwise as well as multiple comparisons are displayed on demand at this stage with a JAVA applet that complies with the JDK 1.1 standard. Alternatively, upstream region sequence and corresponding annotation can be exported in EMBL format (sequence data also in FASTA format). The JAVA applet should run on all JAVA-compatible web browsers. Detailed information about the conserved non-coding block structure are simultaneously shown for multiple upstream regions of different species. If available, annotation information on putative binding sites of transcription factors and EST matches are displayed for the query sequence. The applet facilitates zooming into sequence and annotation. In addition, web links are assigned to sequence features that relate external data sources to the corresponding annotation.

CORG data may be also embedded into other viewers or programs via the distributed annotation system (*DAS*, [30]). DAS facilitates the display of distributed data sources in a common framework with respect to a reference sequence. Our DAS server  constitutes such an external data source. Position information on all conserved non-coding blocks and mapped promoters is accessible from this DAS server. Each DAS sequence feature provides a link to the corresponding CORG database entry. New DAS sources can be easily added to the ENSEMBL display. A small tutorial on installing external DAS data sources is available on our web page .

Additionally, tools for on-site batch retrieval of CORG data will be added to the web portal in the near future.

### Phylogenetic profiling of binding sites

One potential application of CORG is phylogenetic profiling of promoter regions. We define phylogenetic profiling in the context of gene regulation as comparative analysis of presence/absence patterns of binding sites in promoter regions. Here, we consider conserved predicted binding sites and contrast them with validated ones.

#### Serum Response Factor (SRF) promoter

SRF, a MADS-box transcription factor, regulates the expression of immediate-early genes, genes encoding several components of the actin cytoskeleton, and cell-type specific genes, e.g. smooth, cardiac and skeletal muscle or neuronal-specific genes [31,32]. Mouse embryos lacking SRF die before gastrulation and do not form any detectable mesoderm [33,34]. SRF mediates transcriptional activation by binding to CArG box sequences (Consensus pattern: CC(AT)_6_GG) in target gene promoters and by recruiting different co-factors. SRF regulates transcription downstream of MAPK signaling in association with ternary complex factors (TCFs) (for a review see [35]). TCFs bind to ets binding sites present adjacent to CArG boxes in many SRF target gene promoters.

**Figure 2:**
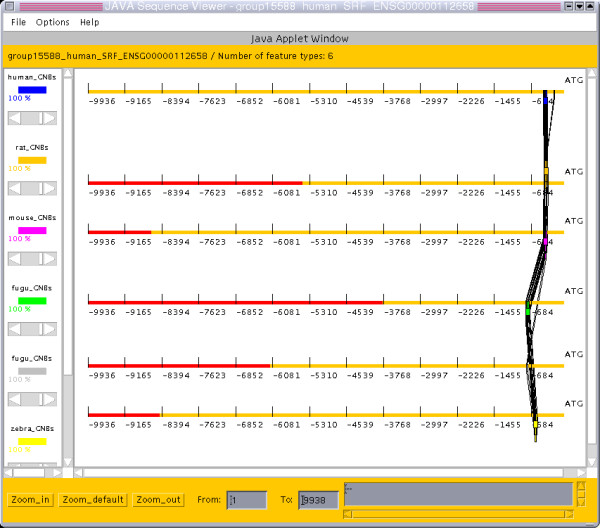
**Graphical multiple alignment view (JAVA applet). ***Multiple alignment view of 6 homologous sequences from 5 species*. All consistent local similarities in the upstream region of SRF homologs are placed relative to the species-specific translation start sites. The distance of the aligned segment to the translation start site is almost equal for all mammals and larger for the fish. The extent of each upstream region is shown as orange bar. Regions covered by flanking genes would be shown in red.

Figure 1 gives an overview of the genomic context of human SRF. As expected, the upstream region of SRF shows substantial conservation to its rodent orthologs. Additionally, significant alignments were found in comparisons with fish homologs (one from zebrafish and two from fugu). The same data is presented in the multiple alignment view of the JAVA applet in Figure 2. This view gives a better idea on the location of alignments in the corresponding source sequences. Note, that the spacing between translation start and alignment is greater in fish than in mammals, which hints at different extension of the promoter region in the two subgroups.

**Figure 3:**
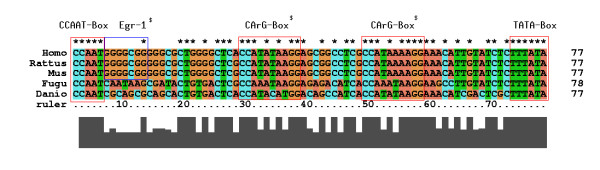
**Textual multiple alignment view**. *Multiple alignment as rendered by CLUSTAL X*. The largest multiple alignment was retrieved from the JAVA applet by a cut and paste operation and rendered in CLUSTAL X [36]. Conserved binding sites are highlighted by red or blue boxes. Known sites as given in TRANSFAC are marked with a dollar sign [42]. Note that the validated Egr-1 site is only conserved in mammals. This site is bound by the serum-inducible Krox-24 zinc finger protein.

We get a better idea on the cause of sequence conservation by browsing the multiple alignment. Textual information can be obtained by clicking on the alignment boxes. Then, the alignment appears in a pop-up window and may be copied to another destination. In Figure 3, we used CLUSTAL X ([36]) to render the conservation structure on to the nucleotide level. Here, a striking observation is the conservation of the regulatory feedback loop of SRF to its own promoter in all species under consideration. So far, this feedback loop was experimentally verified in the mouse system [37] but could exist in all other species under comparison.

### Non-coding RNAs

Non-coding RNA can be classified as transcribed regulatory elements. Non-coding RNAs are also accessible to the user via the CORG database. Since we were primarily interested in non-coding RNAs rather than small mRNA motifs we restricted our search here to long CNBs. A blast search of our multiple alignments with length *L *≥ 50 against the Rfam database [38] and the microRNA Registry [39] identifies 21 alignments as 7 distinct microRNAs and a single snoRNA, Table 2.

**Table 2:** Rfam non-coding RNAs in CORG A + sign indicates that a sequence fragment from the corresponding species (hsa *Homo sapiens*, mmu *Mus musculus*, rno *Rattus norvegicus*, dre *Danio rerio*, tru *Takifugu rubripes*) is contained in the CORG CNB; ∅ indicates that a blast search for an orhologous sequence in the Ensemble database was unsuccessful; n.d. mean no descriptive Ensemble gene annotation. The CNBs containing *mir-196a-2 *are shifted compared to the known microRNA sequences, preventing the detection of the correct stem-loop structure. The B columns marks whether a candidate was identified by a blast search against the Rfam or microRNA Registry, the A column shows whether a hairpin structure was identified by RNAalifold.*p*_RNAz _is the *p*-value for being an evolutionary conserved RNA secondary structure element returned by RNAz.

CNB	B	A	*p*_RNAz_	ncRNA	hsa	mmu	rno	dre	tru	gene
119596	+	+	0.995	mir-34c	+	+	+	+	∅	n.d. (BCT-4)
119607	+	+	0.938							mir-34b in hsa
119658	+	+	0.985							

159914	+	+	0.998	mir-138-2	+	+	+	+	∅	SLC12A3, n.d. in teleosts
159932	+	+	0.999							
159939	+	+	0.998							

194777	+	+	0.998	mir-196b	+	-	+	+	+	HOXA9, dre: HOXA9a and HOXA9b
194820	+	+	0.999							
194839	+	+	0.999							
194941	+	+	0.999							

226470	+	+	0.999	mir-10a	+	+	+	+	+	HOXB4, dre: HOXB4a and HOXB4b
226514	+	+	0.999							
226555	+	+	0.999							
226677	+	-	0.004							

238163	+	+	0.992	mir-10b	+	+	+	+	+	HOXD4, dre: HOXD4a, n.d in tru
238188	+	+	0.984							
238265	+	+	0.994							

391314	+	-	0.125	mir-196a-2	+	+	-	+	+	HOXC9, dre: HOXC9a
391315	+	-	0.999							
391318	+	-	0.511							

470004	+	-	0.218	U93	+	+	+	0	+	n.d.

110374	-	+	0.995	IRES ?	+	+	+	+	+	DGCR8
146100	-	+	0.891		+	+	+	+	0	Ptf1a
393794	-	+	0.999	IRE	+	+	+	+	+	SLCA1

The snoRNA U93 is an unusual mammalian pseudouridinylation guide RNA which accumulates in Cajal (coiled) bodies and it is predicted to function in pseudouridylation of the U2 spliceosomal snRNA [40]. It appears to be specific for mammals. The genomic copy of the human U93 RNA is located in an intron of a series of reported spliced expressed sequence tags (ESTs); furthermore, it has been verified experimentally that U93 is indeed spliced from an intron [40]. It was detectable in the CORG footprint dataset because of its location upstream of a conserved putative gene C14orf87 with unknown function.

The known microRNAs belong to four different groups. The *mir10 *and the *mir196 *precursors are located at specific positions in the *Hox *gene clusters [4-7]. The *mir-196 *family regulates *Hox8 *and *Hox7 *genes, the function of *mir10 *is unknown.

#### Substitution pattern of non-coding RNAs

**Figure 4:**
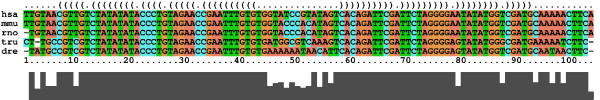
**Alignment and predicted RNA structure of *mir-10b***. The *mir-10b *CNB shows the typical pattern of substitutions in a microRNA precursor hairpin: There are two well-conserved arms, of which the mature microRNA is almost absolutely conserved, and a much more variable loop region. [43].

**Figure 5:**
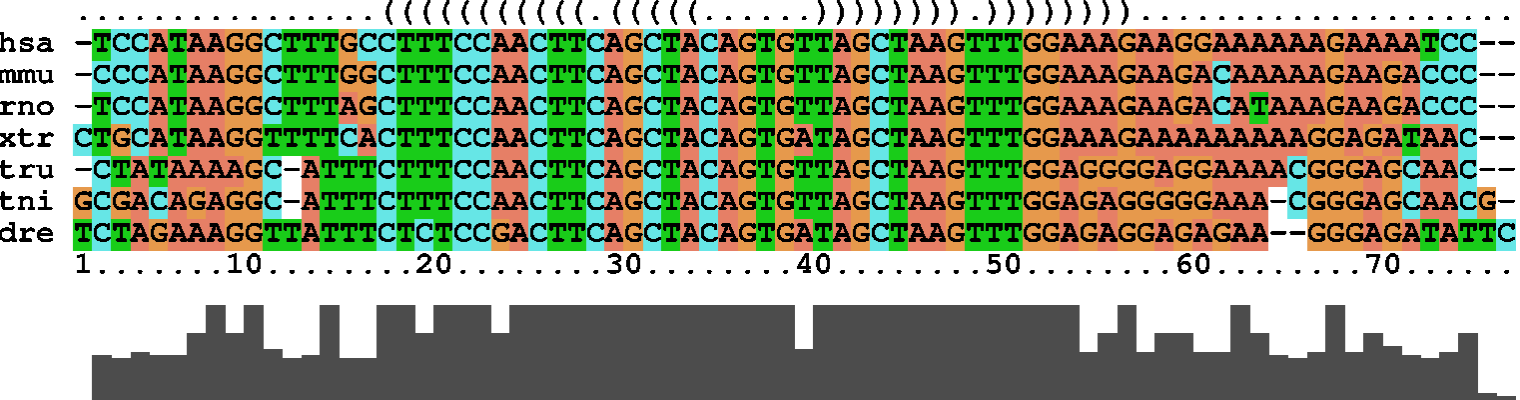
**Alignment and predicted RNA structure of the Iron Response Element**. The Iron Responsive Element (UTRdb [8] identifier: BB277285) shows a substitution pattern that is different from the hairpin structure in Figure 4. Additional orthologous sequences from the frog *Xenopus tropicalis *(xtr), the chicken *Gallus gallus *(gga) and the pufferfish *Tetraodon nigroviridis *are included.

For a microRNA we expect a subsequence of about 20 nt that is almost absolutely conserved among vertebrates (the mature miRNA) and a well-conserved complementary sequence forming the other side of the stem from which the mature microRNA is excised. In contrast, the substitution rate should be much larger in the loop region of the hairpin [41]. *mir10 *is a good example of this typical substitution pattern, which gives rise to a hairpin structure. The pairwise correlation structure of nucleotides is depicted on top of the multiple alignment in Figure 4. A different pattern is observed for the Iron Responsive element in the 5'UTR of *SLCA1*, a member of the sodium transporter family. This time the substitution pattern does not meet the minimal length of the microRNA definition above. Nevertheless, it is conserved across all vertebrate species as shown in Figure 5.

## Conclusion

We have improved and extended our framework of comparative analysis and annotation of vertebrate promoter regions over previous releases (see [20]). The following features have been added to the CORG framework:

• Mapping of validated promoter regions and proper adjustment of the extent of upstream regions.

• Multiple alignments from significant local pair wise alignments.

• Novel approach to predict transcription factor binding sites.

• Web site offers now a genomic context view (as in Figure 1) and an option to export sequence and annotation data.

The CORG database is accessible via our web site. The user is guided step-by-step through the process of selecting and analyzing her promoter region of choice. CORG features an interactive viewer based on JAVA technology, which is tailored to detailed promoter analysis. Large-scale studies make direct use of our DAS service or the MySQL implementation of CORG in conjunction with an application interface (contact authors for details).

We presented selected application examples from the realm of vertebrate gene regulation. Conserved regulatory elements of different kinds (binding sites, microRNAs and UTR elements) are readily accessible to CORG users. New genomes and annotation will be continuously added to CORG.

## Availability and requirements

The database is freely accessible through the website . Programs, scripts and MySQL database dumps are available from the authors upon request.

## Authors' contributions

Christoph Dieterich built the entire pipeline and some parts of the web interface. Steffen Grossmann annotated transcription factor binding sites and provided parts of the web interface. Andrea Tanzer analyzed known and novel RNA elements in the multiple alignments of the CORG database. Stefan Röpcke set up our database of binding site descriptions. Peter F. Arndt worked on an appropriate alignment scoring scheme. Peter F. Stadler and Martin Vingron initiated this work and provided all necessary infrastructure.

## Supplementary Material

Additional File 1 **Distribution of distance between start of transcription and translation**. Histogram of observed genomic distances between start sites of transcription and translation in man for 1,700 entries from the EPD. The red and blue line indicates the 90% and 95% quantiles, respectively. Distances greater than 10^6 ^bp were exluded from the analysis as they mostly occur due to mismappings in the ENSEMBL database. Click here for file
