# Supplementary figures and images for: Comparative promoter region analysis powered by CORG
Source: BMC Genomics. 2005 Feb 21;6:24. doi: 10.1186/1471-2164-6-24 (PMC555765; doi:10.1186/1471-2164-6-24)

## EPD promoter mapping (1700 instances)

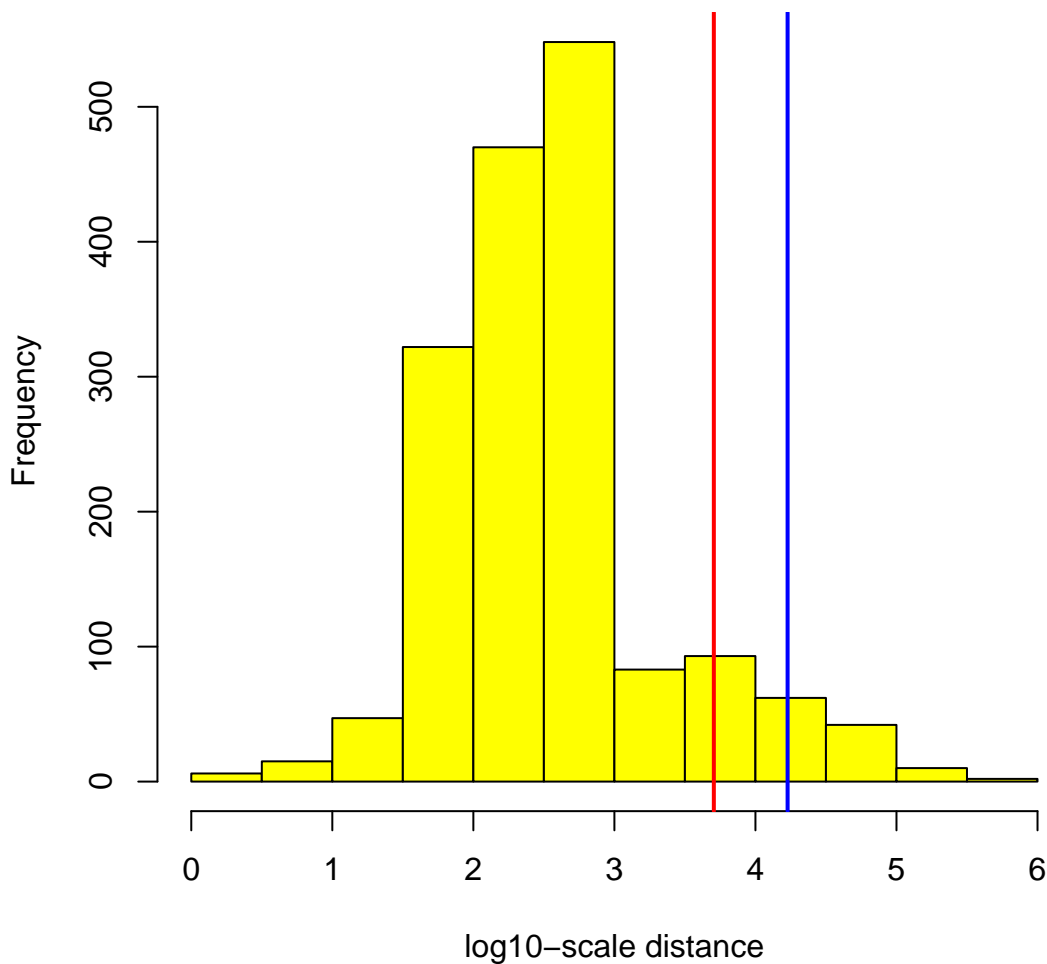

Supplement: Additional File 1 — Distribution of distance between start of transcription and translation. Histogram of observed genomic distances between start sites of transcription and translation in man for 1,700 entries from the EPD. The red and blue line indicates the 90% and 95% quantiles, respectively. Distances greater than 106 bp were exluded from the analysis as they mostly occur due to mismappings in the ENSEMBL database. [file 1471-2164-6-24-S1.pdf]
